# Surface-Plasmon-Assisted Growth, Reshaping and Transformation of Nanomaterials

**DOI:** 10.3390/nano12081329

**Published:** 2022-04-12

**Authors:** Chengyun Zhang, Jianxia Qi, Yangyang Li, Qingyan Han, Wei Gao, Yongkai Wang, Jun Dong

**Affiliations:** School of Electronic Engineering, Xi’an University of Posts & Telecommunications, Xi’an 710121, China; superjianxiaqi@163.com (J.Q.); 18066715534@stu.xupt.edu.cn (Y.L.); hanqingyanlove@163.com (Q.H.); gaowei@xupt.edu.cn (W.G.); ykwang@xupt.edu.cn (Y.W.)

**Keywords:** surface plasmon resonance, hot electron transfer, thermal field, regulation of nanostructures

## Abstract

Excitation of surface plasmon resonance of metal nanostructures is a promising way to break the limit of optical diffraction and to achieve a great enhancement of the local electromagnetic field by the confinement of optical field at the nanoscale. Meanwhile, the relaxation of collective oscillation of electrons will promote the generation of hot carrier and localized thermal effects. The enhanced electromagnetic field, hot carriers and localized thermal effects play an important role in spectral enhancement, biomedicine and catalysis of chemical reactions. In this review, we focus on surface-plasmon-assisted nanomaterial reshaping, growth and transformation. Firstly, the mechanisms of surface-plasmon-modulated chemical reactions are discussed. This is followed by a discussion of recent advances on plasmon-assisted self-reshaping, growth and etching of plasmonic nanostructures. Then, we discuss plasmon-assisted growth/deposition of non-plasmonic nanostructures and transformation of luminescent nanocrystal. Finally, we present our views on the current status and perspectives on the future of the field. We believe that this review will promote the development of surface plasmon in the regulation of nanomaterials.

## 1. Introduction

**Figure 1:**
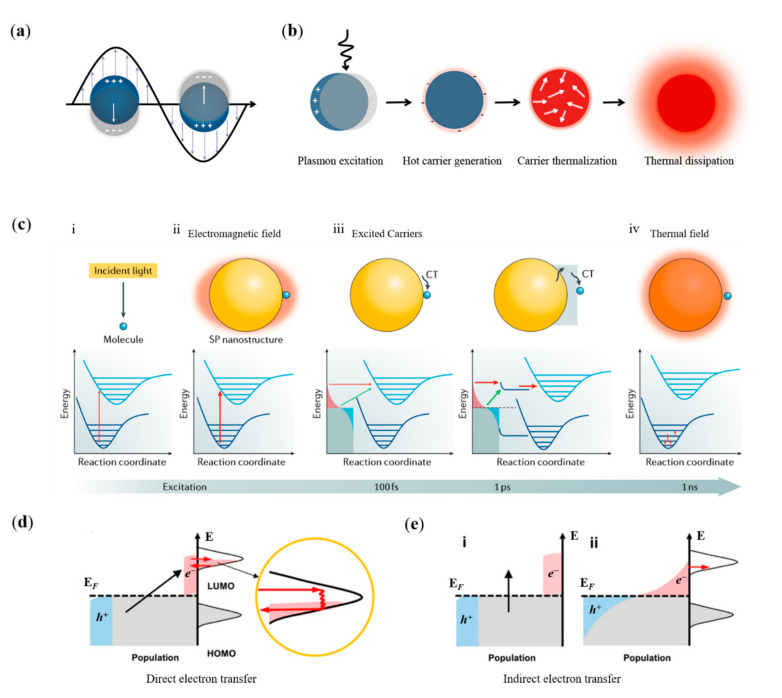
(**a**) Coherent oscillation of electrons and the enhanced EM field [23]; (**b**) Scheme illustration of SPR excitation and relaxation [5]; (**c**) Catalytic effects of photon (i), plasmonic EM field (ii), hot carrier transfers (iii) and plamsonic thermal field (iv) [18]. Direct (**d**) and indirect hot carriers transfer (**e**) between metal nanostructure and reactants adsorbed on the surface [1].

The collective oscillation pattern of conduction band electrons in the nanostructures of certain metals (e.g., Au, Ag and Cu) can effectively enhance the absorption efficiency of the nanostructures, known as surface plasmon resonance (SPR) (Figure 1a) [1,2]. The excitation of SPR can form strong interactions with electromagnetic (EM) radiation and electrons of nanostructures, and lead to a significant enhanced EM field near the nanoparticles (NPs), by converging EM radiation to a size smaller than the wavelength of the incident light [3,4]. The high-energy carriers (electron–hole pairs) and local thermal field will generate during the relaxation of enhanced EM field (Figure 1b) [5,6,7,8]. Since the mid-1970s, plasmonic physics including surface-plasmon-enhanced spectroscopy (Raman scattering, infrared and fluorescence), sensing and waveguiding has been widely studied [9,10,11]. In recent years, the research related to surface plasmon has been extended from plasmonic physics to plasmon-assisted chemical reactions [12,13,14,15,16]. With the enhanced EM field, hot carriers and thermal field, SPR can efficiently modulate surface reactions, especially to enable chemical reactions that are difficult or even impossible to occur under conventional conditions, forming an important frontier in SPR-assisted chemical reactions [17,18,19,20,21,22]. 

High spatial confinement and ultrafast time scale properties of SPR provide a new approach for the precise control of nanomaterial growth and phase transitions. Although plasmonic catalysis on molecules has been extensively studied, systematic reports on the plasmon catalytic effects of nanomaterials are also needed. In this report, we focus on the plasmon catalysis effect on nanomaterials. First, we discuss the mechanisms of plasmon catalysis, including plasmonic EM field, hot carrier transfer and local thermal field. Subsequently, we discuss recent research advances on plasmon catalysis on inorganic nanomaterials, including the reshaping and self-growth/etching of plasmonic nanomaterials, the growth and transformation of non-plasmonic materials. Finally, we present our view on the current state of the field and development in the field of plasmonic-assisted nanomaterial optimization. 

## 2. Mechanisms of Surface-Plasmon-Modulated Chemical Reaction

By photoexcitation of SPR, molecules present in solutions or gas streams or on the surface of metallic NPs can be catalyzed to undergo chemical reactions leading to the deposition of metal atoms or other products, resulting in the growth of nanomaterials. In this process, it is still the reacting molecules that are catalyzed by plasmonic field, hot carriers or thermal field, and the reaction mechanism that steams from a plasmon catalyzed reaction of molecules is applicable in the plasmon-assisted growth of nanomaterials. Therefore, we begin with the discussion of surface plasmon excitation and relaxation, and the mechanism of plasmon catalysis with enhanced EM field, hot carrier transfers and localized thermal field (Figure 1c) [18]. 

### 2.1. The Catalytic Effect of Plasmonic EM Field

For conventional photocatalysis, photon energy excites the electronic transition of the reactants from the ground state to the excited state, and force the molecules to move along the potential energy surface of the excited state (Figure 1c). Finally, molecules in the excited state can either undergo direct chemical reactions or decay back to the ground state with additional vibrational energy, facilitating the overcoming of activation potential [23]. Similar reaction channels of conventional optical excitation are suitable for plasmonic EM field catalyzed reactions [24,25,26,27]. SPR excitation brings about a change in the spatial distribution of the optical field, and eventually the photon density on the surface of metal nanostructures will increase substantially [28,29]. Therefore, the local EM field with higher photon densities will lead to a significantly enhanced reaction efficiency of molecules in the vicinity of nanostructures. For catalytic reactions driven by photon or plasmonic EM fields, resonant energy transfer is required by the energy coupling between the surface plasmon and the reactant molecules. 

### 2.2. The Catalytic Effect of Hot Carriers

SPR in nanostructures can be radiatively relaxed by re-emission of photons or non-radiatively relax by landau damping or chemical interface damping [30,31,32]. If the unoccupied electronic orbitals of the reactance that adsorbed at the interface are coupled to the nanostructure, a new electronic state associated with the chemical bonding at the interface will generate [33,34,35]. The induced electronic states can participate in the coherent oscillations of SPR and accelerate its relaxation, an effect known as interfacial damping [30,36]. During SPR relaxation with the involvement of interfacial damping, hot electrons are generated directly in the orbitals of the electronic unoccupied state of the adsorbates, while hot holes are left in the metal nanostructure (direct electron transfer) (Figure 1d) [1,6]. The electron transfer will produce negative ionic states with instantaneous lifetimes of tens of femtoseconds, which is long enough to allow chemical reactions in the excited state or to increase the vibrational energy of the ground state, thus reducing the reaction potential and facilitating chemical reaction [37]. For this direct electron transfer mechanism, the energies of hot carriers need to overlap with the molecule’s unoccupied state orbitals for the electron exchange to occur. Taking advantage of this feature, the chemical reaction channel can be enhanced selectively by controlling the hot carriers’ energy to achieve a substantial increase in reaction efficiency and selectivity.

The damping effect of SPR due to the energy exchange between the collective coherent oscillation mode of electrons in metals and other particles (e.g., electrons) is called Landau damping [18,38,39]. The energy exchange will accelerate the relaxation of collective coherent oscillations of electrons, and generate hot carriers in metal NPs [40,41,42]. After a thermalization process, a hot Fermi–Dirac distribution formed, then hot carriers with continuous energy distribution are transferred to the orbitals of the adsorbed molecules (indirect electron transfer) (Figure 1e) [1,43]. Since the hot electrons are distributed continuously around the Fermi energy level, the two-step indirect transfer process has a high transfer efficiency and is the most efficient excitation pathway catalyzed by plasmon [44,45,46,47]. The practical efficiency of indirect electron transfer depends on the position of the unoccupied state orbitals of the molecule relative to the Fermi energy level of the metal. The position of the Fermi energy level of the metallic structure is not easily tuned, which means that the energy of thermalized hot electrons is hard to be controlled by the excitation light or the frequency of SPR, thus limiting the ability of plasmon to catalyze selective enhancement of specific chemical reactions.

### 2.3. The Catalytic Effect of Plasmonic Thermal Field

The thermal effect generated by SPR relaxation can be used to heat NPs and surrounding media, and is currently used in photothermal therapy, nanomaterial growth and vapor generation, etc. [48,49,50,51,52,53,54]. In addition, SPR can also reduce the reaction potential barrier and modulate the chemical reactions on the surface of metal [17,55,56,57,58]. Currently, SPR have been used to promote the room temperature dissociation of water, oxygen and hydrogen, as well as the chemical reactions of small molecules on the surface [59,60,61,62].

## 3. Self-Modulatation of Plasmonic Nanostructure

### 3.1. Plasmonic Thermal-Field-Assisted Reshaping of Plasmonic Nanostructure

**Figure 2:**
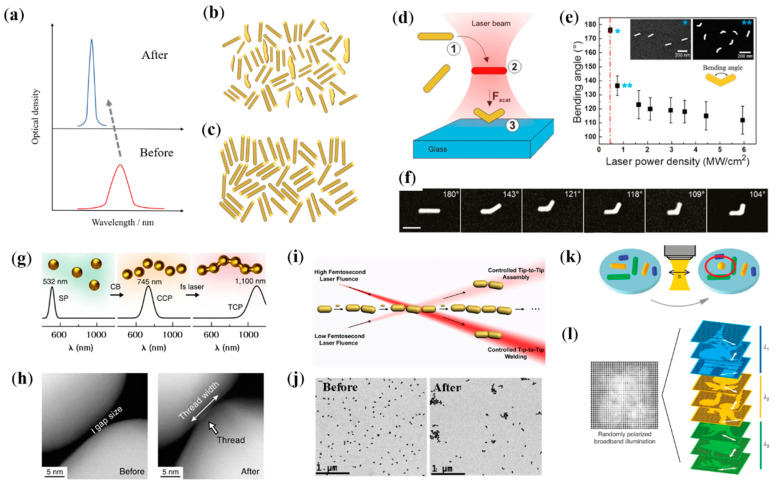
(**a**–**c**) Reshaping of Au Nanorods with the irradiation of femtosecond laser. (**a**) Schematic illustration of optical density spectra of Au Nanorod colloids before (red) and after (blue) laser irradiation. (**b**,**c**) Schematic illustration of Au Nanorods morphology before (**b**) and after (**c**) laser irradiation. (**d**–**f**) Plasmon-assisted bending of Au Nanorods [64]. (**d**) Schematic diagram of optical bending of Au Nanorods. (**e**) Au nanorods transition from a straight (*) to a bent (**) morphology and the control of bending angle with the tune of optical power. (**f**) SEM images of bent Au Nanorods. Scale bar is 100 nm. (**g**,**h**) Plasmon-assisted processing of Au NPs strings [65]. (**g**) Schematic illustration. (**h**) TEM images Au NP chains before (left) and after laser irradiation (right). (**i**,**j**) Plasmon-assisted assembling and welding of Au nanorods [66]. (**i**) Schematic illustration. (**j**) TEM images of Au nanorods before and after assembling. (**k**,**l**) Plasmon-assisted spherification of Au nanorods [67]. (**k**) Schematic illustration of plasmon-assisted spherification of Au nanorods. (**l**) Patterns observed on the detector when irradiated by non-polarized broadband light (left) and light with right polarization and wavelength (right).

The homogeneity of the size and shape of plasmonic metal NPs is the important basis for their unique optical features and has been accepted as a general outcome for judging the quality of the synthesized materials. The reduction of the SPR broadening of colloidal metal NPs that are critically influenced by morphological variations has been a key driver for further progress in nanomaterial colloidal synthesis. Andrés and coworkers report a new method to reshape Au nanorods with plasmonic thermal effect to make their SPR spectra as sharp as those of individual Au nanorods [63]. Femtosecond laser with the same wavelength of SPR band centered at 800 nm was chosen as the source to irradiate Au nanorods. It is found that by precisely controlling the irradiation conditions to balance the relationship between the heat transferred to the surrounding environment and the energy deposited onto the nanorod surface, the nanorods could be effectively reaped and its bandwidth could be significantly reduced (Figure 2a–c). The heat dissipation cooling conditions and the density of surfactants such as CTAB on the surface of Au nanorods are strongly correlated with the shape of the treated Au nanorods. This plasmonic-assisted simple, fast and reproducible method can be used for batch processing of Au nanorods, which is of great importance for its application. Lohmüller and coworkers demonstrated that taking advantage of plasmonic thermal effect, Au nanorods can be bent and reshaped to V-shaped nanoantennas (Figure 2d–f) [64]. It is found that Au nanorods need to be freely dispersed in solution, and it will be bent and printed onto a substrate after light irradiation. With the combination of thermal effect and optical force, Au nanorods will be continuously heated and pushed, finally resulting the melting and reaping. This efficient approach holds great potential for the fabrication of V-shaped antennas in which the bending angle and the orientation of antennas can be independently adjusted by tuning the intensity and polarization of irradiated light. 

In addition to the self-reshape of plasmonic nanostructure, plasmonic thermal effect can also be used to realize the assembly and welding of the nanostructure. Morphological inhomogeneities can lead to a broadening of the SPR of colloidal metal NPs, thus limiting the feasibility of the plasmon-related technological application. As we can in Figure 2g, with the femtosecond laser irradiation Au NPs are connected into a continuous thin thread [65]. Au NPs with plasmon resonance at 532 nm are chemically scaffolded into chains with the use of cucurbit uril (CB) molecules, resulting in resonant redshift to 745 nm. The rigid gaps (about 0.9 nm) of Au NPs that are glued together lead to plasmonic hotspots in gaps and promote transient inhomogeneous distribution of thermal effects (Figure 2h). Au chains are then irradiated with 805 nm pulse laser, resulting in localized threading and the production of strings of Au NPs. The process of plasmon-assisted assembly of Au NPs can be tracked via the variation of optical resonance. Furthermore, plasmon-assisted self-assembly of Au nanorods is realized by irradiation with low flux femtosecond laser (Figure 2i,j) [66]. It is found that the flux of laser pulse is the most important factor that affects the self-assembly kinetics. For nanorod trimers with a longitudinal SPR wavelength of 800 nm (resonating with illumination laser), the number of trimers and longer oligomers is greatly reduced and the relative number of AuNR dimers is increased after irradiation by a femtosecond pulse with a fluence of 100 μJ/cm^2^. If the pulse fluence is greater than 500 μJ/cm^2^, the temperature of the interparticle gaps increases greatly, causing local melting of the Au nanorods tips and thus the Au nanorod is welded. Furthermore, plasmon can also be used to assist the spherification of Au nanorods. As we can see in Figure 2k,l, with the femtosecond laser irradiation the Au nanorods in the focal volume of the focusing objective are reshaped, and the reshaping is selective in the aspect ratio and orientation of the Au nanorods, by taking advantage of the narrow longitudinal SPR linewidth and the dipolar optical response of Au nanorods [67]. Additionally, this longitudinal SPR-mediated selective reshaping is employed to achieve five-dimensional optical information recording and readout.

### 3.2. Hot-Electron-Assisted Self-Growth/Etching of Plasmonic Nanostructure

**Figure 3:**
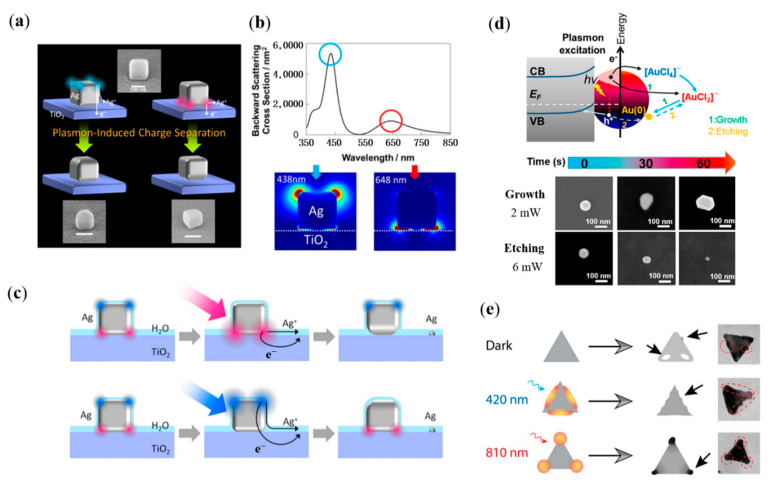
Plasmon-assisted etching of plasmonic nanostructure. (**a**–**c**) Plasmon-assisted site-selective etching of Ag nanocubes [71]. (**a**) Schematic diagram. (**b**) Scattering spectra and EM field distribution of the Ag nanocubes on the TiO_2_. (**c**) Plasmon-assisted electron transition at the interface and site-selective etching of nanocubes. (**d**) Schematic illustration and SEM images of hot carrier-assisted growth/etching of Au NPs [72]. (**e**) Plasmon-assisted spatially controlled replacement reactions on Ag nanoprism [73].

Hot electrons’ generation and transfer during the decay of SPR have attracted much attention recently, which can be applied on plasmonic catalysis, chemical sensing and recording of images based on the oxidation of the NPs [68,69,70]. SPR mode of an Ag nanocube will be divided into distal and proximal mode (resonance modes localized at the top and bottom of the nanocube), when it is placed on a glass plate at different wavelengths. Tatsuma and coworkers realize a site-selective etching of Ag nanocubes, by taking advantage of plasmon-assisted charge separation and transfer (Figure 3a–c) [71]. Hot-electron-assisted oxidation of Ag nanocubes can be controlled by the location of EM field. Additionally, two SPR modes of Ag nanocubes on TiO_2_ localized at the top (438 nm) and bottom (648 nm) of the nanocubes can be selective excited by the wavelength of irradiated light. Therefore, the etching of the top or bottom of Ag nanocubes is optional through switching the wavelength of irradiated light. With the assistance of hot electrons and holes generated with SPR decay, Ding and collaborators realized the controllable growth and etching of Au NPs (Figure 3d) [72]. In their study, Au NPs located on Si substrate are immersed in HAuCl_4_ solution, and 641 nm laser is selected as the irradiation source. When the power of light and the concentration of HAuCl_4_ is relatively low (4 mW, 5 mM), the size of Au NPs is enlarged. When the light with power the of 6 mW and solution with the concentration 20 mM are used, the Au NPs are etched. Plasmonic hot-electron-assisted selective etching is also observed on single Ag nanoprism (Figure 3e) [73]. To investigate the plasmon-assisted spatially controllable chemical reaction, colloidal Ag nanoprisms which SPR mode can be flexibly adjusted throughout the visible region and near-infrared range are chosen. Meanwhile, redox reactions are selected as a model for the chemical reactions of metal particles, which are driven by the differential electrochemical potential, without additional additives. It is found that there is a direct correlation between the excitation intensity and the shift of the in-plane dipole mode, which provides direct evidence for the role of the SPR mode of optical excitation in influencing the galvanic replacement reaction. The spatial control of replacement reactions within the Ag nanoprism depends on the selectively excitation of the SPR mode, which provides a simple and accurate way to design nanomaterials with unique functionality by switching light of external stimulus.

**Figure 4:**
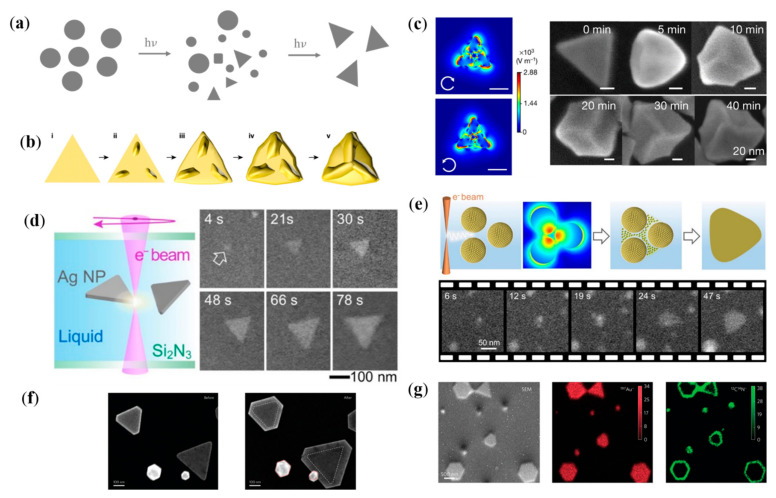
Plasmon-assisted growth of plasmonic nanostructures. (**a**) Schematic illustration of plasmon-assisted conversion of Ag NPs to nanoprisms. (**b**,**c**) Plasmon-assisted synthesis of chiral nanostructure. (**b**) Schematic diagram of chiralization process of Au NPs [76]. (**c**) Calculated EM field of Au NPs under excitation of circularly polarized light and SEM images of Au NPs after laser irradiation for different times. (**d**,**e**) Schematic illustrations and TEM images of plasmon-assisted synthetic Ag nanoprisms [77,78]. (**f**) Morphology of Au nanoprisms before and after plasmon-assisted growth. (**g**) Elemental distribution of Au nanoprisms after plasmon-assisted growth [79].

The plasmonic field and catalysis of hot carriers can be leveraged to synthesize NPs with special shape, and structure. Mirkin et al. report a plasmon-assisted method for converting spherical Ag NPs into triangular nanoprisms by a series of cycles of silver redox [74]. It is the SPR of seed particles that catalyzes the reduction of Ag^+^ to Ag^0^ and induces the conversion to nanoprisms, while Ag^+^ are sourced from the oxidation decomposition of small seed particles by O_2_. The converted colloid shows distinctive optical properties that stems from the nanoprism shape of the Ag NPs (Figure 4a). The size of the converted nanoprisms can be modulated by the frequency of irradiated light. In their subsequent work, they demonstrate that the frequency of irradiated light can be further employed to control defect structure of the plasmonic NPs [75]. Structures with higher number of twin boundaries will be generated, when the irradiated light with higher frequency is used. The correlation between structural defects and irradiated light energy originates from the reduction rate of Ag^+^ that is controlled by the SPR excitation. Chiral inorganic nanostructures with powerful chromophoric activity and self-assembly capabilities have important applications in enantioselective catalysis and optoelectronics. Recently Xu and coworkers propose a plasmon-assisted synthesis method of chiral Au nanostructure, with anisotropy factor up to 0.44 (Figure 4b,c) [76]. They screened a series of chiral ligands and introduced left/right circularly polarized light to the nanoparticle preparation process. By optimizing the wavelength and polarization of the irradiated light, the formation of symmetry breaking on the high index crystal plane is successfully induced and finally obtained chiral nanostructures with homogeneous morphology. This method can be used to achieve the precise synthesis of chiral nanostructures with high optical anisotropy factors. In the works of Sutter and coworkers, surface plasmon is employed to assisted the growth of plasmonic anisotropic nanostructures (Figure 4d,e) [77,78]. With the help of liquid cell electron microscopy, the metal nanoparticle transformation process in solutions containing seed particles is investigated in situ. With the oxidation of the sparsely dispersed Ag seeds, Ag^0^ is uniformly attached to the sides of the nanoprism, making it larger in size. The rate of attachment can be modulated by the intensity of the EM field, as the oxidation reaction of Ag seeds is catalyzed by plasmonic hot carriers. They further investigate the influence of reducing agent and non-aggregation agent to the growth process of Ag seeds. A new growth mechanism is explored whereby aggregation of small Ag NPs in dense suspensions promotes the formation of anisotropic nanoprisms, which coexist and compete with the previously observed direct transformation. The report of plasmon-assisted anisotropic growth of Au nanoprisms demonstrate that the plasmon-assisted growth of Ag NPs is also applicable in the synthesis of other plasmonic metals (Figure 4f,g) [79]. Meanwhile, this work established a fundamental mechanism at the molecular level for the plasmon-assisted self-growth of plasmonic nanostructures.

## 4. Plasmon-Assisted Selective Deposition of Non-Plasmonic Nanomaterials

**Figure 5:**
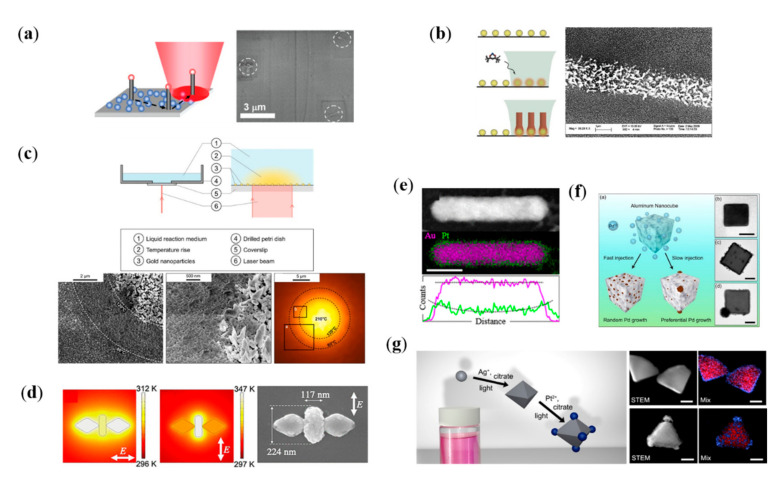
Plasmon-assisted selective deposition of non-plasmonic nanomaterials. (**a**,**b**) Plasmonic thermal-field-assisted selectively chemical vapor deposition. (**a**) Deposition of semiconductor nanowires/Si nanotubes [80] (**b**) Deposition of PbO/TiO_2_ on glass substrates [81]. (**c**) Plasmonic thermal-field-assisted solvothermal synthesis [82]. Schematic illustration (upper panel) and SEM images of synthetic crystal and the corresponding temperature distribution (lower panel). (**d**) Plasmon-assisted growth of ZnO on Au nanoantennas, calculated temperature distributions (left) and SEM image (right) [83]. (**e**–**g**) Plasmon-assisted selective deposition of non-plasmonic materials. (**e**) Deposition of Pt. on the ends of Au nanorods, STEM and EDS map of Pt-Au core shell nanorod [84]. (**f**) Schematic diagram and TEM images of selective dipositive Pd on Al nanocubes [85]. (**g**) Deposition of Pt. on Ag cores. Schematic diagram (left) and TEM, EDS maps of products (right) [86].

In addition to the self-reshaping/growth/etching of plasmonic nanostructures, SPR can be further used to induce the selective deposition of non-plasmonic nanostructures. Plasmonic thermal field with the controllable nanoscale heating capability has great potential to control chemical reactions. Temperatures required for deposition can be easily generated with a tightly focused CW laser beam irradiation on the plasmonic structure. Brongersma, Goodwin and their colleagues have proposed a novel chemical vapor deposition method using the efficient plasmonic local heating strategy and applied it to achieve selective deposition of many types of nanomaterials, such as semiconductor nanowires, Si nanotubes, PbO nanowires and TiO_2_ polycrystalline dots (Figure 5a,b) [80,81]. In this method, a substrate with plasmonic nanomaterials is exposed to a gaseous environment that contains reactant precursor. Then, with the resonance light irradiation, non-plasmonic nanomaterial growth can be observed in the illuminated NPs. The high spatial control and energy-efficient properties of the plasmon-assisted chemical vapor deposition method inspires new pathways for the building of novel photothermal devices. With Au NPs as photothermal sources, a plasmonic thermal-field-assisted solvothermal synthesis is proposed, that can be used to achieve spatially controlled deposition of a wide range of materials, as long as solvothermal synthesis exists (Figure 5c) [82]. Similarly, Sasaki and coworkers proposed a plasmon thermal-field-assisted hydrothermal synthesis, and realized the targeted location of ZnO on the surface of Au nanoantennas, with the help of a confined heat production function at the nanoscale (Figure 5d) [83]. 

Then, we discuss plasmon-assisted location of non-plasmonic nanomaterials on the tips of plasmonic structures with the more concentrated EM fields. With the longitudinal SPR excitation, plasmonic hot electrons as the redox agent catalyzed the nucleation of Pt^0^ in the presence of chloroplatinic acid (Figure 5e) [84]. The anisotropic spatial distribution of hot electrons promotes the selective deposition of platinum onto Au nanorods. In the work of Halas and coworkers, monodisperse Al nanocubes with sharp corners are obtained. Taking advantage of plasmon catalysis of Al nanocrystals, they further realized an Al-Pd heterometallic NPs, by regioselective deposing of Pd nanoclusters on the vertices of nanocubes (Figure 5f) [85]. Similar strategy is used by Personick et al., and the reduction of Pt. ions onto Ag cores is realized (Figure 5g) [86]. Ag-Pt core−shell and core−satellite structures are successfully synthesized by controlling the Pt. ion reduction rate with the excitation of SPR.

## 5. Plasmon-Assisted Transformation of Luminescent Nanocrystal

Rare-earth ion-doped inorganic luminescent nanomaterials are widely noticed and applied because of their rich linear energy level structure, full spectral coverage of fluorescence emission function in the UV-Vis-IR region and stable physicochemical properties [87,88]. In general, matrix materials with single crystal structures are required for high selectivity and polarizable photoemission. For micro/nano systems, subsequent annealing treatment is generally used to achieve the optimization of the crystal structure and improvement of physicochemical stability and luminescence properties. However, the conventional annealing process tends to cause the agglomeration and uncontrollable morphology of particles, and it is hard to realize selective optimization of the particles. Technical bottleneck of conventional method is an urgent problem in the field of nanocrystal growth and luminescent materials research.

**Figure 6:**
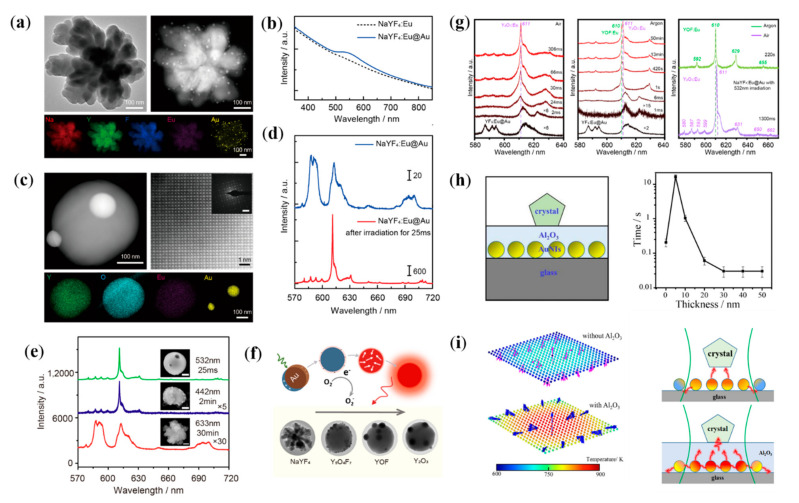
Plasmon-assisted crystal transformation from polycrystalline fluoride to single crystal oxide. (**a**,**b**) TEM, EDX maps and absorption spectra of NaYF_4_:Eu and NaYF_4_:Eu@Au; (**c**) STEM, EDX and SAED maps of transformed single-crystalline Y_2_O_3_ NP; (**d**) Fluorescence spectra of NaYF_4_:Eu and NaYF_4_:Eu@Au and transformed Y_2_O_3_:Eu. (**e**) Fluorescence spectra and SEM images of transformed products after light irradiation with different wavelengths of light. (**f**) Schematic diagram of plasmon-assisted polycrystalline fluoride NPs transformation. (**g**) Evolution of luminescence spectra during the plasmon-assisted crystal (YF_3_:Eu@Au) transformation in air (left) and argon (middle), and luminescence spectra of YF_3_:Eu particle after annealed at 600 °C for 1 h (right) [90]. (**h**) Schematic diagram of heat trapping structure (left) and the required irradiation time for the crystal transformation with different thickness of dielectric layer Al_2_O_3_ (right). (**i**) Simulated thermal transfer modes (left) and schematic diagram of the mechanism (right) of pure Au nanoislands and Au nanoislands/Al_2_O_3_ systems, the direction of the arrow representing the direction of heat flow [91].

Recently, our group has developed a new method for nanocrystal optimization and achieved rapid and controllable nanocrystal transformation by the thermal effect of surface plasmon and the catalytic effect of plasmonic hot electrons [89,90,91,92]. Polycrystalline rare-earth-doped fluoride NPs prepared at room temperature were chosen as the main target for the study (Figure 6a). The process and mechanism of the transformation of rare-earth crystals driven by surface plasmon were investigated by using the luminescence of trivalent rare-earth ions as the probe of structure and in situ characterization techniques [89]. The results show that the transformation of polycrystalline NaYF_4_ to single-crystalline Y_2_O_3_ NPs can be achieved under the irradiation of visible light at the milliwatt level and within milliseconds thanks to the efficient light absorption of Au NPs (Figure 6b,c) [89]. The optimization of crystal structure and crystallinity of the products leads to a significant increase in fluorescence radiation intensity, red–green ratio and monochromaticity (Figure 6d). Then, the effects of resonant and non-resonant excitation of surface plasmon on the crystal transformation and the synergistic mechanism of interband absorption of Au NPs on the crystal transformation are investigated. The results show that the thermal effects and hot electrons generated during the interband transition of Au NPs are lower in energy compared with the resonant excitation process of SPR, resulting in significantly lower crystal transformation rates and poor crystallinity of products under 442 nm laser irradiation (Figure 6e). The kinetic processes of crystal structure change are also investigated. The results show that the thermal effect and hot electrons generated during the relaxation of SPR act synergistically to promote the crystal particle transformation. The thermal effect of the surface plasmon drives the optimization of the crystal structure and induces the transformation from polycrystalline to single crystal (Figure 6f,g) [90]. Meanwhile, the hot electrons catalyze the oxidation process of fluoride to oxide by interacting with oxygen molecules to generate O_2_^−^ ions with strong oxidation properties. 

To enhance the utilization of plasmonic thermal energy, we designed a heat trapping structure by introducing a dielectric layer Al_2_O_3_ to the surface of self-assembled Au nanoislands (Figure 6h,i) [91]. The absorption cross section of light gradually increases with the thickening of the Al_2_O_3_ layer, and the heat is mainly transferred to the dielectric layer Al_2_O_3_ due to the increase in the effective refractive index. In addition, the Al_2_O_3_ layer transfers a large amount of heat generated by the more Au NPs around the laser spot to the crystal. Both the enhanced light absorption and heat utilization result in higher temperatures and faster crystal transformation rates. When the thickness of Al_2_O_3_ is too thin (5–10 nm), the heat diffuses through the Al_2_O_3_ layer into the surrounding environment, resulting in greater heat loss and slower crystal transition efficiency. Overall, these results suggest that a heat-trapping structure with a sufficiently thick Al_2_O_3_ can ensure effective light absorption and heat utilization, thus improving the efficiency of the SPR-assisted photothermal conversion. For the transformation of luminescent nanomaterials, the direct catalytic target of surface plasmon is inorganic nanocrystals rather than molecules, which opens up a new field of plasmon-assisted catalysis.

## 6. Conclusions

In this review, we give an overview of surface plasmon excitation and relaxation, and the mechanism of plasmon catalysis with enhanced EM field, direct/indirect hot carrier transfers and localized thermal field. Meanwhile, we have introduced recent advances of plasmon-assisted modulation of inorganic nanomaterials including the plasmonic nanomaterials and other non-plasmonic nanomaterials. Especially, the application of surface plasmon in the rapid in situ achievement of luminescent single crystal NPs is reviewed in detail.

Related effects of surface plasmon resonance have many potential and irreplaceable advantages in inducing nanocrystal reshape/growth/transitions, and the related experimental and theoretical studies are of great significance for the acquisition of high-quality nanomaterials and the applications of surface plasmon. Although a lot of results have been achieved in the existing works, there are many shortcomings that need to be improved and explored subsequently. For example, although the kinetics of carrier relaxation processes in bulk metals have been extensively studied, these time scales may change in nanomaterials due to confinement effects, and therefore further studies of hot carrier relaxation kinetic processes in nanosystems are needed. Secondly, plasmonic hot carriers have a strong catalytic effect, but the low transfer efficiency, short lifetime of carriers and the energy distribution that cannot be easily regulated have limited the application of carriers in regulating the structure of nanomaterials. Moreover, plasmonic metal nanostructures have strong photothermal conversion efficiency, but it is difficult to achieve a large and stable temperature gradient on the metal surface due to the fast diffusion of heat from the high thermal conductivity of the metal. Thus, it limits the spatial selectivity of plasmonic thermal field catalysis. We believe that once the hot carriers and local temperature distribution are effectively controlled, the field of plasmonic catalysis will achieve great success in assisting the highly tunable and selective growth and transformation of nanomaterials.
